# Insights into the Internalization and Retrograde Trafficking of Dengue 2 Virus in BHK-21 Cells

**DOI:** 10.1371/journal.pone.0025229

**Published:** 2011-10-04

**Authors:** Nidhi Shrivastava, Samatha Sripada, Jasmine Kaur, Paresh S. Shah, D. Cecilia

**Affiliations:** National Institute of Virology, Pune, India; Institut Pasteur, France

## Abstract

**Background:**

Dengue virus (DENV) enters cells via endocytosis, traffics to perinuclear (PN) region, the site of morphogenesis and exits by exocytosis. This study aims to understand the role of dynamin II, endosomes, microtubules (MT) and dynein in the early events of DENV replication.

**Findings:**

Using double immunoflourescence labelling of DENV-2 infected BHK-21 cells it was observed that the surface envelope (E) protein of the virion associated with dynamin II from 0–30 min post infection (p.i.). The sphincter like array of dynamin II supported its pinchase-like activity. The association with endosomes was observed from 0 min at cell periphery to 30 min in the perinuclear (PN) region, suggesting that internalization continued for 30 min. Association of E protein with alpha-tubulin was observed from 8 h indicating that it was the newly translated protein that trafficked on the MT. Dynein was found to associate with the E protein from 4 h in the cytoplasm to 48 h in the PN region and dissociate at 72 h. Association of E protein with dynein was confirmed by immunoprecipitation. Overexpression of dynamitin, which disrupts the dynein complex, resulted in loss of trafficking of viral E and core proteins. The findings corroborated with the growth kinetics assessed by quantitation of viral RNA in infected BHK-21 cells. The detection of E protein at 4 h–8 h correlated with detectable increase in viral RNA from 8 h. The detection of high concentrations of E protein in the PN region at 24–48 h coincided with release of virus into the supernatant starting from 36 h p.i. The dissociation of dynein from E protein by 72 h was coincident with maximum release of virus, hinting at a possible negative feedback for viral protein translation.

**Conclusion:**

The study shows for the first time the association of dynamin II with DENV-2 during entry and dynein dependent retrograde trafficking of DENV proteins on microtubules.

## Introduction

Dengue is by far the most devastating of all mosquito borne viral diseases, caused by dengue virus (DENV), a member of the family *Flaviviridae*. More than 3 billion humans live in dengue endemic regions of the world and currently more than 50 million infections occur annually with at least 500,000 individuals requiring hospitalization [1]–[3]. Development of antiviral agents has so far focused on inhibiting viral enzymes, i.e. nonstructural protein 3 (NS3) [4]. An understanding of virus morphogenesis and protein-protein interactions could provide new targets for intervention. DENV is an enveloped virus with a positive sense single-stranded ∼11 kb RNA genome [5]. Infection begins with attachment of virus particles to host receptors, several of which have been identified as putative receptors i.e. heparan sulphate [6], heat shock proteins, Hsp70 and Hsp 90 [7], Glucose-regulated protein (GRP78/Bip) [8], a 37-kDa/67-kDa high affinity laminin receptor [9], [10] and Dendritic cell-specific intercellular adhesion molecule-3-Grabbing non-integrin (DC-SIGN) [11]. After binding to the receptor DENV undergoes either clathrin mediated endocytosis [12], or directly enters into the cytoplasm of cells by fusion at the plasma membrane (PM) [13], [14]. Following internalization, the virus envelope undergoes fusion with the endosomal membrane due to low pH, and the viral nucleocapsid is released into the host cytoplasm [15], [16]. The viral RNA is then translated to yield structural and nonstructural viral proteins followed by transcription and replication of RNA, packaging and maturation in the perinuclear (PN) region [17] and egress by exocytosis [18] or budding at the PM [19]. The entire process of morphogenesis is dependent on the movement of virus and viral components within the cell. The cytoskeleton is an integral component for intra-cellular trafficking [20], [21] with all 3 components, the microfilaments, the microtubules (MT) and the intermediate filaments, involved [22]. Dynein, is a microtubule associated motor protein and is responsible for the movement of cargo from cell periphery to cell centre, from the endoplasmic reticulum (ER) to the Golgi [19]. A crucial role for dynein in the MT associated transport of viral proteins has been reported for hepatitis C [23], African swine fever [24], Hanta [25], polio [26] and rabies [27] viruses. Although the major events in morphogenesis are broadly understood for DENV, the detailed events underlying, entry and retrograde trafficking have not been entirely delineated. The present study uses confocal microscopy to visualize internalization, endocytosis and early trafficking of DENV-2 proteins and shows for the first time association of dynamin II with the envelope (E) protein during entry and relevance of dynein in the retrograde trafficking of envelope and core (C) proteins.

## Materials and Methods

### Cells and Virus

BHK-21 (Baby Hamster Kidney) (C-13, American Type Culture Collection) cells were used as the cell line was suitable for both infection as well as transfection studies. The cells were maintained in Minimal Essential Medium (MEM) with 10% fetal bovine serum (FBS) at 37°C and 5% CO_2_, supplemented with 200 U/ml of penicillin/streptomycin and 1% glutamine. All cell culture reagents were procured from Gibco-BRL. DENV-2 (803347) was obtained from the Institute's Virus Repository and used in all the experiments.

### Antibodies and reagents

For immunofluorescence assay primary antibodies included mouse monoclonal antibody (MAb) against DENV-2 E glycoprotein and C protein (a kind gift from Dr. Askov, Queensland University of Technology, Australia), goat antibody against dynamin II, mouse MAb against dynein intermediate chain (Santa Cruz Biotechnology Inc., CA, USA), mouse MAb against alpha tubulin (Sigma, USA). Secondary antibodies used were goat anti-mouse IgG conjugated to FITC or donkey anti-mouse IgG conjugated to Alexa 488, donkey anti-goat IgG conjugated to Alexa 594 (Molecular Probes, Eugene, OR, USA) and rabbit anti-mouse IgG conjugated to TRITC (Sigma). Lysotracker Red (Molecular Probes) was used to stain endosomes.

### Immunofluorescence Assay

BHK-21 cells were seeded one day prior to infection at a minimum concentration of 1×10^5^ cells/ml per 55 mm petri dish (Tarsons) containing 9×22 mm cover slips. Cells were infected with DENV-2 at a multiplicity of infection (MOI) of 1 or 5. Adsorption was carried out for 1 h at 4°C to prevent virus entry and synchronize infection of cells. Cells were washed twice with cold medium to remove unbound virus and replenished with prewarmed medium. The cover slips were taken out of the culture at various time points post infection (p.i.) under sterile conditions, washed with 0.01 M phosphate buffer saline pH 7.2 (PBS) and fixed in ice-cold acetone for 10 min at −20°C for experiments with dynein. Cells labeled for dynamin II and endosomes were fixed with 3.7% paraformaldehyde for 30 min at room temperature (RT), followed by permeabilization with 0.1% Triton X-100 for 2 min and quenching with 0.01 M NH_4_Cl. The fixed cells were washed with PBS and blocked with 1% bovine serum albumin (BSA) in PBS for 30 min. For dual staining, viral E or C proteins were labeled with specific mouse MAbs and the cellular components with the specific antibodies as mentioned in Table 1. The primary antibodies to viral protein and cellular components were added simultaneously to the cells and incubated for 1 h followed by washing with PBS. This was followed by incubation with appropriate secondary antibodies conjugated to fluorescent dyes, FITC, TRITC, Alexa 488 or Alexa 594 (Table 1) added simultaneously. DAPI (4′, 6′ diamino-2-phenylindole) was used to stain nucleus in all experiments. At the end of the staining process, cover slips were washed and mounted onto slides using mounting medium MOWIOL (Calbiochem, CA, USA). Control cells, which were not infected but submitted to the same procedures, were included in all experiments as mock infected cultures.

**Table 1:** Primary and secondary antibodies used for staining viral and cellular proteins.

Protein	Primary antibody	Conjugate
Dynamin II	Goat anti-dynamin II (1∶20)	Donkey anti goat IgG Alexa 594(1∶250)
Endosomes	Lysotraker Red (5 ug/ml)	—
Alpha tubulin	Mouse anti-alpha tubulin (1∶100)	Rabbit anti-mouseIgG TRITC(1∶400)
Dynein	Mouse anti-dynein (1∶100)	Donkey anti-mouse IgG Al*exa 488* (1∶400)
E protein	Mouse anti-E MAb (1∶10)	Rabbit anti-mouse IgG TRITC(1∶400)Goat anti-mouse IgG FITC (1∶200)Goat anti- mouse IgG Alexa 488 (1∶400)
C protein	Mouse Anti-core MAb (1∶10)	Rabbit anti-mouse IgG TRITC(1∶400)

### Transfection with GFP-dynamitin plasmid

The GFP-dynamitin expressing plasmid was a kind gift from Dr. Beate Sodeik, Hannover Medical School, Germany. BHK-21 cells, grown on coverslips in 6 well plates, were transfected with 2 ug GFP-dynamitin plasmid using Lipofectamine 2000 Plus (Invitrogen) according to manufacturers instructions. Briefly, 2 ug of plasmid was diluted in 250 ul of MEM mixed with 250 ul of MEM containing 10 ul of Lipofectamine and incubated for 30 min at RT. The DNA-liposome complex was then added to preformed monolayer of BHK-21 cells with 70% confluency in serum free medium and incubated at 37°C. Cells were then infected with DENV-2 (MOI of 1) at 6 h post transfection. The cells were fixed after 36 h p.i. and stained for DENV-2 E or C protein using mouse anti-E or anti-C MAb followed by anti-mouse IgG labeled with TRITC. For comparison mock-transfected cells were infected with DENV-2 and processed similarly.

### Image acquisition and image analysis

The images were acquired using Laser Scanning Microscope 510 META (Carl Zeiss, Germany) with 40× and 63× oil objective lens corrected for oil immersion. For dual color analysis, green and red emissions were recorded sequentially through appropriate filters (505–530 band-pass filters for Alexa 488/FITC and 560 nm long-pass filters for Alexa 594/TRITC). Post acquisition image analysis was done using the 3D projection option of LSM software, surface rendering option of Huygens Essential (Scientific Volume Imaging, The Netherlands) and maximum intensity projection using Imaris (Bitplane Scientific software). Three-dimensional (3D) images, which cover the entire depth of the cell, were derived from deconvolved images. All voxels (volume pixels) in the image with a given colocalization level were joined, forming a 3D surface. In the surface rendering option of the software, the surface is split into different closed unconnected parts enabling independent analysis.

### Coimmunoprecipitation assay

BHK-21 cells were infected with DENV-2 (MOI of 1). At 36 h p.i., the cells were washed with ice cold PBS (0.01 M), scraped and centrifuged at 2000 rpm. The cells were lysed in 5 pellet volumes of ice-cold lysis buffer (50 mM Tris-HCl,150 mM NaCl, 0.5% (v/v) NP40, 0.1 mM NaF, 10 mM DTT and protease inhibitor cocktail). The clarified cell lysate containing approximately 500 ug of protein was incubated with 10 ul of equilibrated Sepharose protein A beads for one hour at 4°C for preclearing and centrifuged to recover lysate. The precleared lysate was incubated overnight at 4°C with human anti-DENV IgG (purified from a serum sample that had plaque reduction neutralization titre of 12,500) for formation of immune complexes. The immune complexes were captured by Sepharose protein A beads by incubating for 3 h at 4°C, centrifuged and washed three times with lysis buffer. The beads were resuspended in 30 ul of sodium dodecyl sulfate (SDS) sample buffer (125 mM Tris, pH 6.8, 4% SDS, 20% glycerol), boiled for 5 min, centrifuged and the supernatant was loaded on 12.5% polyacrylamide gel under non reducing conditions. The proteins were transferred to nitrocellulose membrane for Western blot analysis. The blot was probed with anti-dynein antibody followed by goat anti-mouse IgG labeled with Horse radish peroxidase (HRP) (Sigma) and developed with diamino benzedene substrate. Mock infected BHK-21 cell lysate pulled down with Sepharose protein A beads served as negative control. Infected BHK-21 cells lysate was used as positive control.

### Real time PCR

Viral RNA was quantitated in the infected cultures using a two step real time RT-PCR test reported previously [28]. BHK-21 cells were infected at MOI of 1 and culture supernatant and cells were harvested at different time points starting from 0 h to 120 h p.i.

## Results

### Kinetics of DENV-2 replication in BHK-21 cells

**Figure 1:**
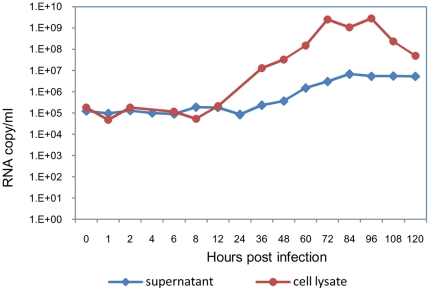
Growth curve of DENV-2 in BHK-21 cells. BHK-21 cells were infected with DENV-2 (803447) at 1 MOI and culture supernatants and cell lysates were collected at regular intervals from 0–120 h p.i. Viral RNA was quantitated by real time RT-PCR.

Prior to undertaking experiments on entry and early intracellular trafficking, the kinetics of viral RNA production in BHK-21 cells was determined. BHK-21 cells were infected with DENV-2 at MOI of 1 (∼10^9^ RNA molecules) and the cultures were assessed for cell-associated and cell free viral RNA at different time points p.i. by real time RT-PCR (Fig. 1). Despite washing off the unadsorbed virus, viral RNA (10^5^ molecules) was detected in cells as well as culture supernatant, from 0–6 h after infection. Similar levels of viral RNA at early time points after infection were shown in a previous report [29]. It is possible that virus particles, which are not internalized, remain loosely bound, and are detectable for a long time in culture. The inefficiency of the process of infection for flaviviruses with a PFU: particle ratio of 1∶4000 has been reported before [30], [31]. The cell-associated viral RNA levels showed a rise from 12 h onwards till 72 h (10^9^ molecules) and then decreased to 10^7^ particles by 120 h. Viral RNA in the supernatant started increasing from 36 h and peaked at 84 h (10^7^ molecules) after which the level reached a plateau till 120 h. The cultures could not be tested after 120 h due to cell lysis.

### Association of dynamin II with DENV-2 E protein

**Figure 2:**
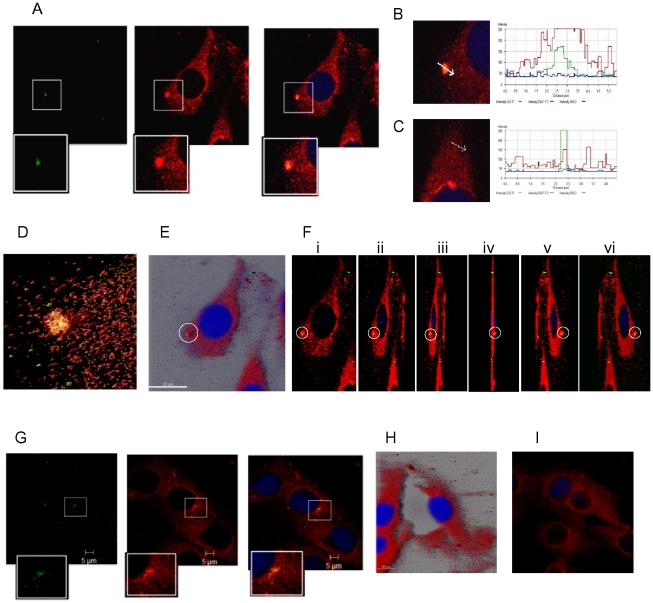
DENV-2 associates with dynamin II during internalization. BHK-21 cells were infected at MOI of 5. The cells were fixed at different time intervals and stained for DENV-2 with mouse anti-E MAb and for dynamin II with goat anti-dynamin II antibody, followed by donkey anti-mouse IgG Alexa 488 and donkey anti-goat IgG Alexa 594. The images of the E-dynamin complex analyzed using different programs are shown; **A**] split images with magnified insets at 0 min, **B**] intensity profile at 0 min showing points of colocalization and **C**] no colocalization **D**] 3D deconvolved image of optical sections of 2.5 µm using Huygen's essential software **E**] Maximum Intensity projection (MIP) highlighting the surface location of the complex (Imaris 7.0) **F**] 3 D projections at different angles (i) 60°, ii) 90°, iii) 100°, iv)110°, v) 120° and vi) 130° using Carl Zeiss AIM 4.0. **G**] split image at 30 min and **H**] no association at 1 h (Imaris 7.0). **I**] Mock infected cells showing the presence of dynamin II.

Dynamin II, a MT associated GTPase protein is known to regulate clathrin-mediated endocytosis [32], [33]. The association of dynamin II with DENV-2 E protein and the duration of the association were determined to understand the kinetics of internalization. BHK-21 cells were infected with DENV-2 at MOI of 5 to ensure infection of most cells simultaneously. Virus adsorption was carried out on ice to synchronize internalization [34]. After adsorption virus was removed and the monolayer was washed with cold medium. Immediately after adding pre-warmed medium, cells were fixed and processed for staining after 0, 5, 10, 15, 30, 60 and 120 minutes. Cells were double stained for DENV-2 with mouse anti-E MAb and for dynamin II with goat anti-dynamin II antibody, followed by donkey anti-mouse IgG-Alexa 488 and donkey anti-goat IgG-Alexa 594. The E protein was presumed to represent the incoming virus, as it is the major surface protein on the virion [5]. Colocalization of E protein and dynamin II was observed from 0 min to 30 min p.i. The colocalization foci were counted and analyzed in 20 fields at 0, 5, 15 and 30 min time points (Table 2). The number of cells showing the presence of colocalization foci decreased from 50% at 0 min to 17% at 30 min. The size of the foci did not vary with time. The size of foci ranged from 0.75 um to 2 um, within which the spot of green fluorescence representing virus was 0.2–1 um (size of virus 50 nm) suggestive of each focus representing an aggregate of virus associating with dynamin II. In the area of colocalization the average fluorescence intensity for dyanmin II was 234 and did not change with time. The intensity of fluorescence for E protein decreased from 145 at 0 min to 84 at 15 min. The pattern of intensity reversed at points of no colocalization with average fluorescence intensity of 48 for dynamin II and 207 for E protein. The colocalization of E protein with dynamin II is depicted in the split image (Fig. 2A). The intensity of DENV E protein at a point of no colocalization (Fig. 2B) is higher than that observed in the area of colocalization (Fig. 2C) as shown by the intensity profiles. For a clearer depiction of the merging of virus and dynamin II, deconvolved images of the cell in Fig. 2A were generated using Huygens surface rendering (Fig. 2D) wherein free virus can be seen (green) around the focus of colocalization (yellow). To show that the complexes were present on the surface, z-stack images of the infected cells were acquired and analyzed by Maximum Intensity Projection (Imaris) (Fig. 2E), which clearly shows the location of the aggregate on cell surface. Rotation of the image along its y-axis and 3D imaging using Zeiss software (Fig. 2F) further confirms the surface location of the complex. The colocalization between E protein and dynamin II decreased by 30 min p.i. (Fig. 2G). At 60 min (Fig. 2H) there was no association between E protein and dynamin II. Fig. 2I shows the distribution of dynamin II in mock infected cells. The results illustrate the involvement of dynamin II in the process of virus internalization which peaked in the first 5 min of infection.

**Table 2:** Characterization of foci of colocalization of E protein with dynamin II.

Time points	Percentage of cells with foci	Size of foci	Peak intensity of Alexa 594 (Dynamin II)	Peak intensity of Alexa 488 (DENV-2 E protein)
0 min	50	1.4 (1–2)*	238^@^	145^@^
5 min	38	1.43 (0.75–2)	252	116
15 min	19	1.3 (1–2)	210	84
30 min	17	1.3 (1–2)	238	99

*Average size (range) in µm.

@ Average of the peak intensity observed in the focus of colocalization.

### Trafficking of virus within endosomes

**Figure 3:**
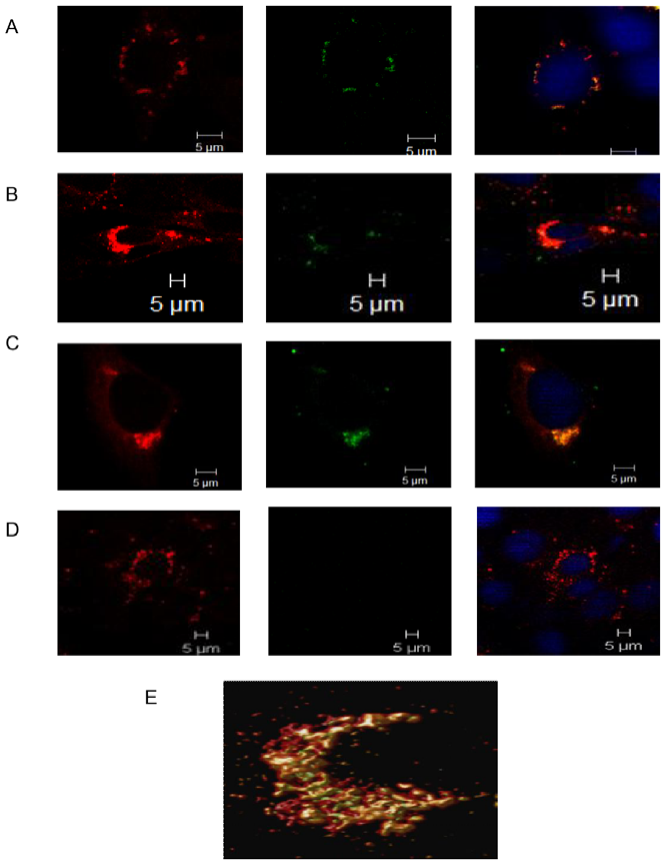
DENV-2 E protein traffics within the endosomes post internalization. BHK-21 cells were infected at MOI of 5. Cells were stained for endosomes by adding lysotracker red to the cells 2 h prior to fixation. The cells were stained for DENV-2 E protein using mouse anti-E MAb followed by goat anti-mouse IgG FITC. Split images show DENV-2 E protein associating with endosomes at **A**] 0 min **B**] 30 min **C**] 2 h and **D**] 4 h. **E**] 3D reconstruction of the z stacked (thickness 3.30 µm) deconvolved image for the 30 min time point.

Lysotracker Red dye was used to follow the kinetics of DENV-2 association within endosomes. Lysotracker red is an acidophilic dye which selectively stains low pH containing compartments and would therefore detect DENV-2 in both early and late endosomes Cells were infected with DENV-2 at 5 MOI, similar to the experiment with dynamin II and stained for E protein and endosomes at 0 min, 10 min, 15 min, 30 min, 1 h, 2 h, 4 h, and 8 h post infection. Lysotracker red was added to cells 2 h prior to fixation and cells were labeled for E protein using mouse anti-E MAb followed by goat anti-mouse IgG FITC after fixing. Tracking the movement of endosomes showed that the endosomes positive for E protein, were visible close to cell periphery at 0 min p.i. (Fig. 3A), were present throughout the cytosol within 15 min and collected in the PN region by 30 min (Fig. 3B). By 2 h (Fig. 3C) only a few cells showed association and by 4 h there was no colocalization (Fig. 3D). The close association was further confirmed by the deconvolution analysis of the 30 min image (Fig. 3E). The number of E-positive endosomes observed was higher than the number of dynamin II-E protein colocalization foci per cell, reflecting the transient nature of dynamin II-E protein complexes. The results indicate that DENV-2 traffics within endosomes and is delivered to the PN region within 30 min. Infected cells were labeled for the presence of viral C protein at 1 h, 2 h and 4 h p.i. to detect capsid released within the cells. Absence of positive signal indicated that the concentration of released capsid was below detectable levels. The decrease in E protein-positive endosomes by 2 h indicates end of entry events.

### Association of DENV-2 E protein with microtubule (MT)

**Figure 4:**
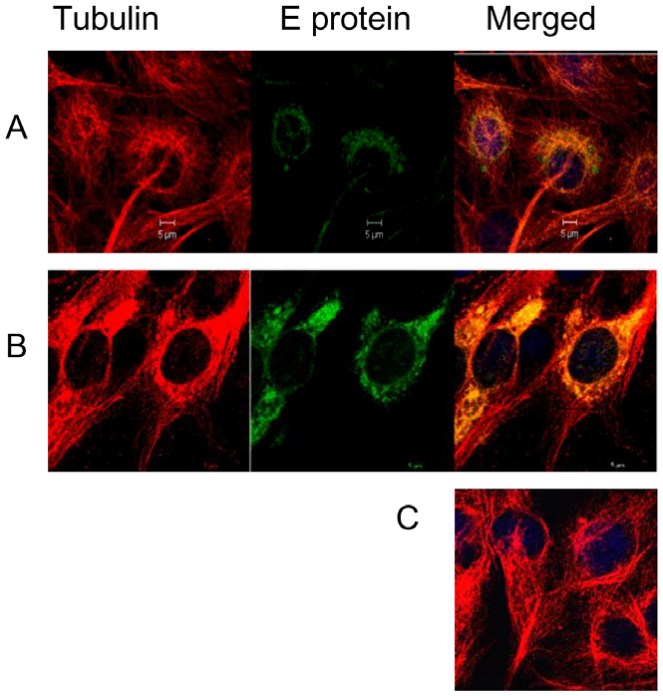
DENV-2 E protein traffics on microtubules. BHK-21 cells infected at 1 MOI were fixed at different time points and stained for DENV-2 E protein and anti-alpha tubulin. The E protein was labelled with mouse anti-E MAb followed by goat anti-mouse IgG FITC conjugate. Cells were saturated with unlabeled goat anti-mouse IgG and then stained with mouse anti-alpha tubulin MAb followed by rabbit anti-mouse IgG TRITC. Infected cells showing colocalization of DENV E protein and alpha-tubulin, **A**] at 8 h and **B**] at 24 h. **C**] Mock infected cells showing distribution of alpha-tubulin.

The microtubule network is the major highway along which there is movement of endocytosed material. Colocalization of the E protein with alpha tubulin was therefore examined in BHK-21 cells infected at 1 MOI. Infected cells were fixed at different time points, 1 h, 2 h, 4 h, 8 h and 24 h p.i. The fixed cells were labeled first with mouse anti-E MAb followed by goat anti-mouse IgG FITC. The labeled cells were saturated with unlabeled goat anti-mouse IgG, then stained with mouse anti-alpha tubulin MAb followed by rabbit anti-mouse IgG TRITC. Close association between the two proteins was observed from 8 h (Fig. 4A) and maximized at 24 h (Fig. 4B) especially in the PN region, representative of the microtubule organization centre (MTOC). Mock infected cells are shown in Fig. 4C. No colocalization was observed at early time points prior to 8 h p.i. Although confocal microscopy has its limitations, the observation of E protein associating with microtubules from 8 h onwards and not prior to that suggested that microtubules are intimately involved in trafficking of newly synthesized E protein and not the entering virions.

### Dynein dependent retrograde trafficking of DENV-2 E protein

**Figure 5:**
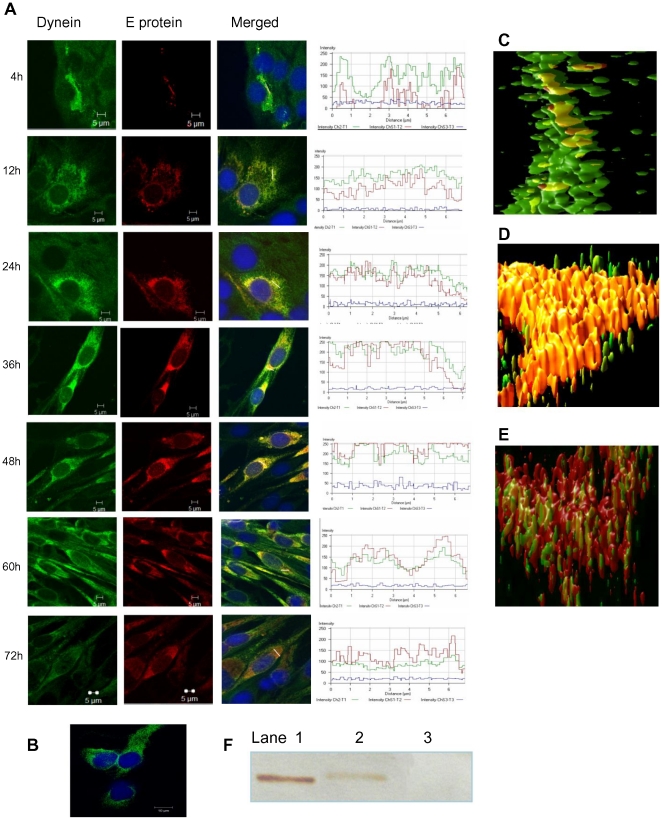
DENV-2 E protein associates with dynein during retrograde trafficking. BHK-21 cells infected with DENV-2 at 1 MOI were fixed at different time points p.i. DENV-2 E protein was stained using mouse anti-E MAb followed by rabbit anti-mouse IgG TRITC, saturated with goat anti-mouse IgG and then stained for dynein using mouse anti-dynein MAb followed by donkey anti-mouse IgG Alexa 488. **A**] The split images and the intensity profiles showing the colocalization are displayed for each of the time points; 4 h, 12 h, 24 h, 36 h, 48 h, 60 h and 72 h. **B**] Mock infected control cells stained for DENV-2 E protein and dynein. **C**] The 3D reconstruction of the z stacked deconvolved images showing the beginnings of association of E-dynein at 4 h, followed by **D**] strong colocalization at 48 h and then **E**] dissociation at 72 h. **F**] Co-immunoprecipitation of DENV-2 E-dynein complex. Lysates of DENV-2 infected or uninfected BHK-21 cells were co-immunoprecipitated with anti-DENV IgG antibody and the blot was probed with anti-dynein antibody followed by goat anti-mouse HRP. Lane 1 - DENV-2 infected. BHK-21 cell lysate, Lane 2 - Immunoprecipitated DENV-2 infected BHK-21 cells, Lane 3 -Immunoprecipitated uninfected BHK-21 cells.

Dynein is a minus-end directed motor protein complex, which is implicated in the trafficking of viral proteins towards the PN region [25], [35]. DENV morphogenesis is known to occur in the PN region in the endoplasmic reticulum (ER) and Golgi apparatus [17], [36]. To determine whether dynein was the motor protein involved in MT based trafficking of DENV-2 E protein, infected cells were fixed and doubled stained for dynein intermediate chain and viral E protein at different time points from 2 h to 72 h p.i. For E protein and dynein, both the primary antibodies were mouse derived therefore the labeling was done sequentially with an intervening step of blocking. After reacting the cells with mouse anti-E MAb followed by rabbit anti-mouse IgG TRITC, the cells were blocked with unlabeled goat anti-mouse IgG. Cells were then incubated with mouse anti-dynein MAb followed by donkey anti-mouse IgG Alexa 488. There was no E protein visible at 2 h (not shown). Panel 5A shows the pattern of E-dynein association at 4 h, 12 h, 24 h, 36 h, 48 h, 60 h and 72 h with the intensity profiles for both fluorochromes in the marked region of colocalization. Dynein in uninfected cells was distributed evenly in the entire cell (Fig. 5B). In the infected cell, intensity of dynein increased around the E protein. At 4 h, the E protein was present at very low concentration and colocalized with dynein. As time progressed, the concentration of E protein increased and by 12 h, a discrete bead like pattern of E-dynein complex, was seen which corresponded to the distribution of ER suggestive of trafficking to and from ER [37]. At 24–48 h maximum concentration of the dynein-E complex was observed in the PN region. Association of viral protein in the Golgi apparatus has been reported before [38]. At 60 h, colocalization was weak and by 72 h, dissociation between dynein and E protein was observed. Another facet of infection was revealed by the intensity profiles accompanying each split image. In infected cells the concentration of E protein and dynein were inversely proportional to each other as infection progressed indicating the requirement of dynein for early trafficking of E (intensity profiles in Fig. 5A). Deconvolved images were used to generate 3D reconstruction of E-dynein at 4 h (Fig. 5C), 48 h (Fig. 5D) and 72 h (Fig. 5E), which depicted the association followed by dissociation of dynein with E protein as infection progressed. The kinetics of association of dynein with E protein suggests that the newly synthesized E protein uses dynein for retrograde trafficking to the site of assembly and dissociates from it at 72 h when maximum virus is present in the cell supernatant (Fig. 1).

### Co-immunoprecipitation of dynein with DENV-2 E protein

Co-immunoprecipitation was used to prove that the colocalization between E protein and dynein observed by confocal microscopy represented formation of E-dynein complex. BHK-21 cells were infected with DENV-2 and lysed at 36 h p.i., a time point at which maximum colocalization was observed. IgG, purified from the serum of a dengue immune individual was used to immunoprecipitate the proteins from infected and uninfected cell lysates. The immune complexes resolved by SDS-polyacrylamide gel electrophoresis (PAGE) under non reducing condition followed by Western blot were identified with antibody directed against the intermediate chain of dynein (Fig. 5F). The reactivity of anti-dynein antibodies with cell-associated dynein is shown in Lane 1. The presence of dynein in the immune complex co-precipitated with human anti-dengue IgG coated sepharose A beads is visible in Lane 2. There was no signal in the immunoprecipitated uninfected BHK-21 lysate in Lane3. The results of the co-immunoprecipitation assay validated the colocalization of E protein-dynein visible in infected cells by confocal microscopy.

### In silico docking of E protein with dynein

**Figure 6:**
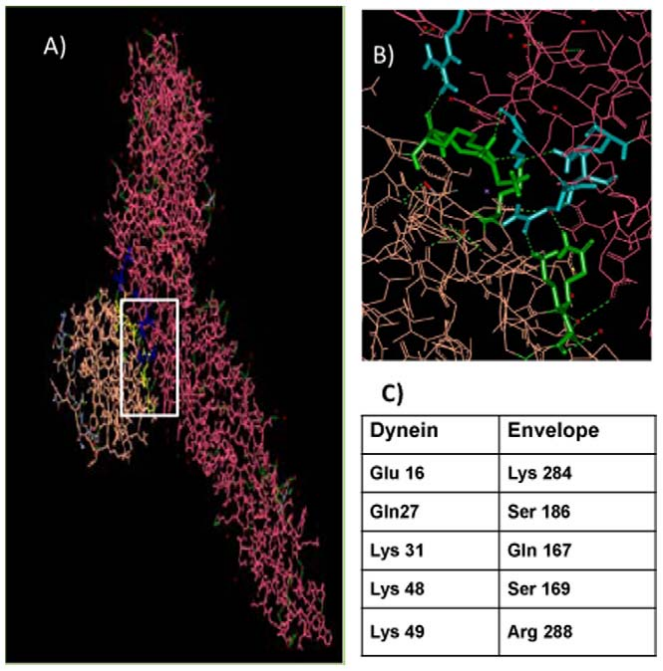
Docking of DENV-2 E protein with dynein. The E protein monomer [PDB:1TG8] was docked with dynein light chain (LC8) [PDB:2P2T] using Hex 5.1 version. **A**] The carbon chain backbone models of the two proteins docked together, E protein (pink) and dynein (orange). **B**] The interacting residues of E protein (blue) and dynein (green) **C**] list of interacting residues on E protein and dynein.

The light chain of dynein (LC8) bound to residues 128–138 of intermediate chain (IC74) is reported to contain the cargo binding site [39] of the MT- dependent motor protein complex. Hex (Version 5.1), which is a molecular superimposition and docking program based on 8 Dimensional fast Fourier transform (FFT), was used to carry out docking between the E protein of DENV-2 and dynein using structures downloaded from the Protein Data Bank (PDB). DENV-2 E monomer (PDB:1TG8) was docked with dynein (PDB:2P2T). The model with the lowest energy was selected for further analysis using Accelrys Discovery Studio visualizer (version 2.5). Five residues on the dynein molecule were found to interact with five residues on the E protein (Fig. 6A,B). The five residues identified in the E protein (Fig. 6C) were conserved in 87 other strains of DENV-2 (protein sequences downloaded from Genbank) when analyzed by Clustal W2 (MEGA 5.03). All five residues were located in domain I of the E protein but were discontinuous. Whether the residues are actually involved in binding will require further studies.

### Loss of Dynein motor activity by over expression of Dynamitin

**Figure 7:**
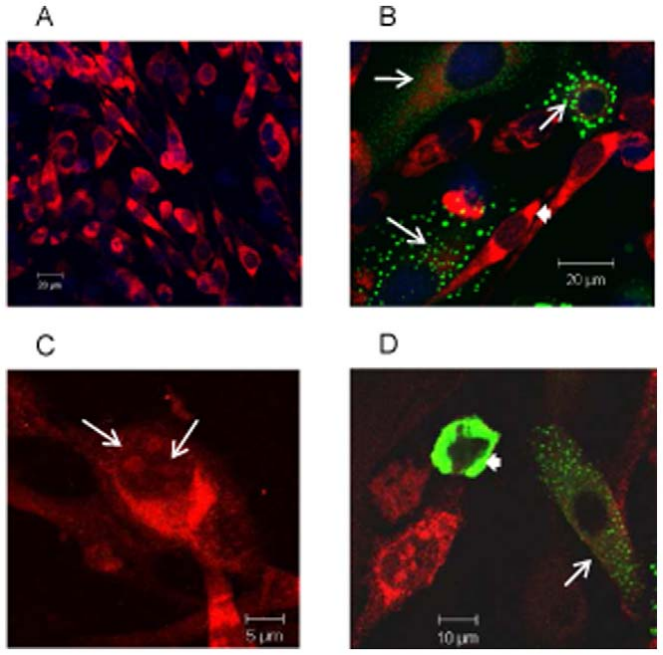
Over expression of Dynamitin inhibits trafficking of DENV-2 E and core proteins. BHK-21 cells transfected with GFP-dynamitin expressing plasmid were infected with DENV-2 at 1 MOI. The cells were fixed after 36 h p.i. and stained for DENV-2 E protein (A, B) or C protein (C, D) with mouse anti-E or anti-core MAb followed by anti-mouse IgG TRITC. **A**] Control non-transfected infected cells **B**] Cells transfected with GFP-dynamitin, and infected 6 h post transfection showed low levels of E protein (white arrows) in dynamitin expressing cells compared to the high intensity of E protein (white arrowheads) in dynamitin negative cells. **C**] Non transfected infected cell showing nuclear localization of core protein (white arrows). **D**] Cells showing low levels of core protein in cytoplasm (white arrows) in GFP-dynamitin expressing cells. One cell with high expression of GFP-dynamitin shows complete absence of infection (white arrowhead).

Dynein along with its cofactor dynactin, mediates MT-dependent retrograde trafficking. Over expression of p50/dynamitin, a subunit of dynactin acts as a dominant-negative inhibitor of dynein-dynactin interaction which blocks dynein mediated transport [40], [41]. The effect of dynamitin over expression on DENV-2 E protein trafficking was investigated. Cells were transfected with GFP-dynamitin and infected with DENV-2 at 6 h post transfection. The infected cells were stained for E protein at 36 h p.i., the time point at which maximum colocalization of E protein with dynein had been observed. In control nontransfected infected cultures, >90% of the cells were infected and positive for E protein which accumulated in the PN region (Fig. 7A). In transfected infected cultures GFP-dynamitin positive cells showed low expression of E protein in the cytosol and not in the PN region as seen in adjacent dynamitin-negative cells (Fig. 7B). Therefore there was inhibition of expression of E protein and its trafficking to the PN region. The effect of over-expression of dynamitin on the transport of C protein was also investigated. DENV C protein is known to contain nuclear localization signals, and is transported to the nucleus [42]. In the infected mock transfected cells C protein was observed in the cytosol and in the nucleus (Fig. 7C). In comparison in GFP-dynamitin transfected infected cells, the C protein was expressed but restricted to the cytosol (Fig. 7D). Cells showing high intensity of GFP-dynamitin (white arrowhead) had no detectable C protein. A similar pattern was also seen for the E protein (not shown). The results thus unequivocally proved the role of dynein in trafficking of DENV-2 E and C proteins.

## Discussion

The current study was undertaken to determine the interactions of cellular proteins with E protein during the internalization and trafficking of DENV-2 in mammalian fibroblast cells (BHK-21) using confocal microscopy. DENV has been shown to gain entry into different cell types using different modes of internalisation, i.e. clathrin/caveolae dependent endocytosis [12] and fusion at PM [14]. We studied the dynamin II dependency of virus entry. Dynamin II belongs to a family of GTPases associated with formation of nascent vesicles and promotion of vesicle fission [43] and is closely associated with clathrin-mediated endocytosis. We were successful in showing a strong association between DENV-2 E protein and dynamin II using confocal microscopy. The E protein is the major surface glycoprotein of DENV, which is responsible for engaging the receptor [44], [45]. Therefore visualising the E protein in the early steps of virus entry was considered representative of tracking the virion. We used the E-dynamin II association to determine the kinetics of DENV-2 entry. Colocalization of DENV-2 E protein with dynamin II within seconds was in concordance with the function of dynamin II, which during clathrin mediated endocytosis aggregates around the vesicle and serves as pinchase-like mechanoenzyme to facilitate the formation of endocytic vesicles by severing nascent endocytic pits from the PM [46]. The higher intensity of dynamin II at the foci of colocalization was perhaps a reflection of dynamin II aggregation at the site of endocytosis [47]. The observation of lower intensity of E protein in the foci of colocalization compared to the points of no colocalization could be due to lower accessibility of the E protein to the antibody once the virus had entered the process of endocytosis. The presence of large amounts free virions on the cell surface was supported by the detection of substantial viral RNA at 0–6 h p.i. by real time RT-PCR. This kind of visualization of the endocytosis process is being shown for the first time. The maximum internalization occurs within the first 15 min of infection, however the E-dynamin II complex could be visualized at the cell surface till 30 min. This indicates that DENV-2 internalization is a process that goes on till 30 min p.i. The kinetics with confocal microscopy was in agreement with the internalization kinetics reported earlier by electron microscopy [6] and estimation of internalized infectious virus by PFU assay [48], where maximum internalization of virus occurs within the first few minutes of infection.

Labelling for endosomes and E protein revealed that DENV-2 localised in endosomes within 0 min p.i. and by 30 min the E protein positive endosomes were observed mostly in the PN region. Therefore internalization and the process of virus trafficking to the PN region via endosomes continue for 30 min. Association of dengue virus with early and late endosomes has been shown at 3 min and 17 min post infection respectively by live cell imaging [49], [50]. The absence of endosome free E protein in the cytoplasm during this period indicated that virus travels to the site of replication protected in the endocytic vesicles and uncoating occurs directly at the site of replication. Following endocytosis the low pH of the endosomes results in trimerization of the envelope protein [51], [52] followed by fusion and uncoating of nucleocapsid. It has been reported for other flaviviruses that most of the steps in replication occur in the PN region [53]. Intracellular shuttling of both proteins and vesicles requires cytoskeletal filaments and molecular motor proteins [54], [55]. Staining for MT and DENV-2 E protein showed close association between the two from 8 h onwards, with viral protein accumulating in the PN region. Cytoplasmic dynein transports cargo toward the microtubule minus end. Co staining with dynein revealed colocalization of DENV-2 E protein with dynein from 4 h p.i. Before this time point the E protein could not be seen either in association with dynein or independent. The last sighting of E protein was at 2 h within endosomes. Transcription of viral RNA has been reported to occur by 2 h [49]. It follows therefore that uncoating and the first round of translation of genomic RNA should be occurring before 2 h. The fact that the E protein could not be observed in the cells till 4 h indicated that the concentration of the initially translated protein is too low to be detected by immunofluorescence assay. Therefore the E protein was seen colocalized with dynein only after the *de novo* synthesis of viral proteins had reached a substantial concentration. By 12 h most of the dynein was engaged by the viral E protein. The E-dynein complex aggregated to the PN region by 36 h, which is now known to be the region of high viral activity [17], [53]. Further proof of binding of E protein to dynein was provided by co-immunoprecipitation at 36 h post infection and also by protein-protein docking analysis. A putative site of interaction comprising of five residues was identified on the E protein. Three of the five residues are present in the recognition sequence on dynein-binding cargo proteins. Dynein mediated transport of the E protein on the microtubules is perhaps involved in the movement of E protein to the ER and from the ER to the Golgi apparatus, the major players in DENV morphogenesis [5]. The loss of association by 60–72 h was co-incidental with maximum release of virus into the supernatant of infected cultures as proved by real time RT-PCR data. Loss of association with dynein indirectly suggests reduction in synthesis of E protein. Therefore, it is possible that when there is peak virus production, there is shutdown of viral protein synthesis.

To prove that dynein mediated trafficking was essential for DENV-2 replication, dynein motor activity was disrupted by over expression of dynamitin and its effect was seen on the expression of viral E protein. Dynamitin, a subunit of dynein dynactin complex, is required for cargo transport [41]. Over expression of dynamitin resulted in lower concentrations of viral E and C proteins and inhibited their translocation to target sites. Higher expression of dynamitin resulted in total inhibition of E and C protein expression, which was indicative of inhibition of virus replication. Thus dynein was crucial to the trafficking of newly synthesized DENV structural proteins, E and C.

In conclusion the virions gain entry via dynamin II assisted endocytosis. They are translocated from cell periphery to PN region within endosomes. The newly translated DENV-2 protein binds to dynein and traffics on MT to PN region which is known to be the site of assembly. The association of dynein in the intracellular transport of DENV-2 proteins is being shown for the first time.
